# Fight outcome briefly affects the reproductive fitness of male crickets

**DOI:** 10.1038/s41598-018-27866-4

**Published:** 2018-06-26

**Authors:** Yang Zeng, Feng-Hao Zhou, Dao-Hong Zhu

**Affiliations:** Laboratory of Insect Behavior and Evolutionary Ecology, Central South University of Forestry and Technology, Changsha, 410004 Hunan Province China

## Abstract

Sexual selection allows male individuals to adopt different evolutionary strategies in mating system. In this study, we determined whether dominance affected reproductive fitness of male crickets *Velarifictorus aspersus* during both pre-copulatory and post-copulatory selection when we excluded male–male competition. The results showed that females mated more often with male winners only during the first 2 h after a fight when male winners were more likely to produce courtship songs than losers. However, females did not retain the attached spermatophores of male winners longer than those of male losers, and the fecundity and fertilization success also did not differ significantly between females mated different times with male winners and losers. Instead, the fertilization success was positively correlated with male body weight. These results suggest that a recent wining experience increases reproductive fitness of males during pre-copulatory selection, but females may prefer larger males rather than winners during post-copulatory selection. The incoordination between pre- and post-copulatory selection may allow males to adopt different evolutionary strategies in mating system.

## Introduction

Sexual selection is one of the most powerful force in determining the reproductive success of all individuals. Darwin has proposed that sexual selection operates in two mechanisms: male-male competition and female mate choice^1,2^. Because these two mechanisms can work simultaneously on the same species, males may adopt different evolutionary strategies in mating system. For example, in Giant hissing cockroaches *Gromphadorhina oblongonota*, some males invest heavily in weapons which allows them to be more successful in male-male competition, but some others invest heavily in testes development which may give them advantages in sperm competition^3^.

Fighting behavior is common among male animals. The fighting behavior comprises a sequential escalating series of behaviors, which ends when one male surrenders. The outcome of a fight determines the ownership of resources and it can also dramatically change the subsequent behavior of males. For example, male crickets that win a fight can become more aggressive and have a greater chance of winning another fight, whereas losers become non-aggressive and tend to lose other fights^4,5^. This phenomenon is known as the “winner–loser” effect and it has also been reported in many other species, where it usually last from minutes to hours^6,7^.

Male winners usually have a greater chance of copulating by suppressing the male losers^8^, suggesting that dominance positively affects competition for access to mating partners. However, it is unclear whether male dominance affects the mating choice of females. Some studies have shown that the male winners of fights are the preferred mates because they are of higher quality^9^. However, some studies found no correlation between male fighting ability and attractiveness^10,11^. Sexual selection continues post-copulation if the females mate multiple times, which is known as sperm competition. For example, female crickets retain the attached spermatophores of preferred males longer than those of less desirable males, thereby resulting in greater sperm transfer and increased fertilization success^12–15^. The post-copulatory selection can act to reinforce pre-copulatory selection^16^ or oppose it^17,18^.

The fighting behavior of male crickets is highly impressive and it has been studied extensively^19–21^. The burrowing cricket *Velarifictorus aspersus* Walker (Gryllidae) distributes widely in China. Males of this species have extremely large mandibles and they often fight with other males using their mandibles when competing for burrows and females^22^. A previous study showed that the courtship behavior of male losers was suppressed by the presence of male winners, and that females mated more often with male winners than losers, thereby suggesting that males can increase their reproductive fitness by winning a male–male competition^22^. In the present study, to determine whether male dominance affects reproductive fitness of male crickets during both pre-copulatory and post-copulatory selection when male–male competition is excluded, we investigated the mating behaviors of males and females when male winners and losers were kept separately with a sexually mature female at different times after fighting. We also compared the spermatophore retention time, fecundity, and fertilization success of females mated with winners or losers to determine whether male dominance affected the post-copulatory reproductive fitness.

## Results

### Effect of dominance on the courtship behavior of males

**Figure 1:**
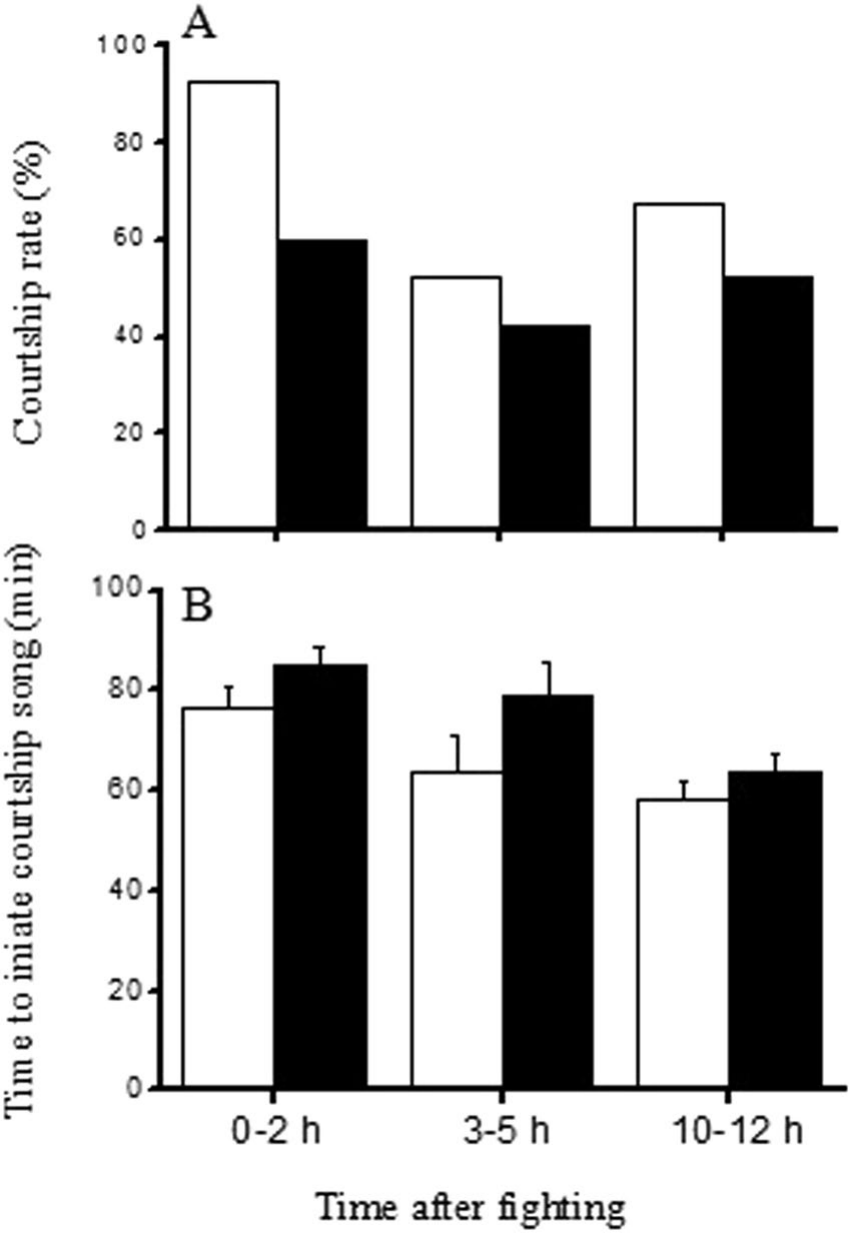
Comparison of the courtship rate (**A**) and time before singing was initiated (**B**) for winners and losers at different times after the end of fighting (empty bars: winners, solid bars: losers; n = 40).

During the first 2 h after fighting, more than 90% of the male winners produced courtship songs, whereas only 55% and 67.5% of male winners produced courtship songs during 3–5 h and 10–12 h after fighting, respectively. Among the losers, 60%, 47.5%, and 50% of males produced courtship songs during the periods 0–2 h, 3–5 h, and 10–12 h after fighting, respectively. Courtship rate was significantly affected by fight outcome and the time after fight (GLM with binomial errors, fight outcome: χ^2^ = 12.517, *P* < 0.001; time after fight: χ^2^ = 14.841, *P* < 0.001). Male losers were less likely to produce courtship songs than male winners during the first 2 h after fighting, but not during 3–5 h and 10–12 h after fighting (Fig. 1A). Male winners and losers required more than 60 min to initiate courtship songs, and there were no significant differences between winners and losers (Two-way ANOVA, fight outcome: df = 1, F = 3.700, *P* = 0.056). However, males took longer to produce courtship song after a recent fight than they did if the fight was 10-12 hours prior (Two-way ANOVA, time after fight: df = 2, F = 6.089, *P* = 0.003) (Fig. 1B).

### Effect of dominance on female mating behavior

**Figure 2:**
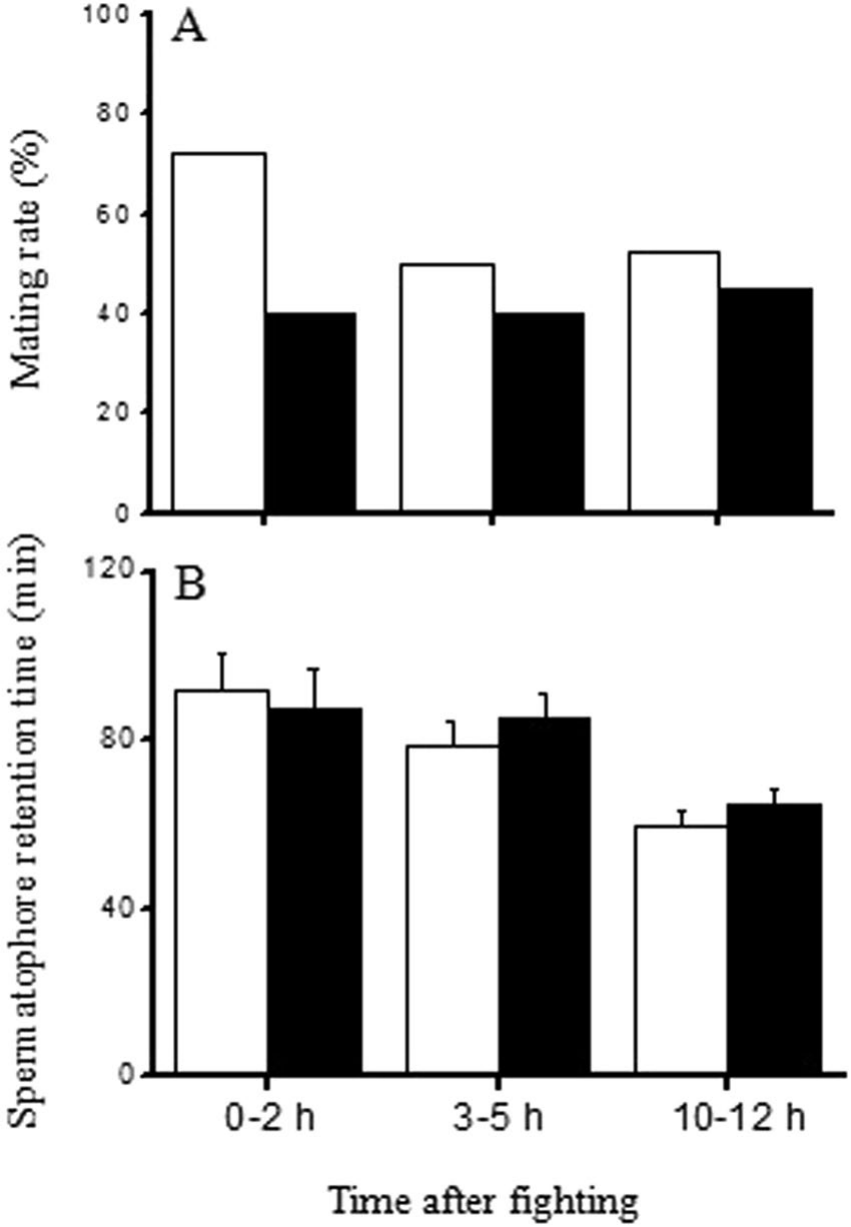
Comparison of the mating rate (**A**) and spermatophore retention time (**B**) in females that mated with winners or losers at different times after the end of fighting (empty bars: winners, solid bars: losers; n = 40).

During the first 2 h after fighting, females mated with more male winners than male losers, whereas they mated with equal numbers of male winners and losers during the periods 3–5 h and 10–12 h after fighting (GLM with binomial errors, fight outcome: χ^2^ = 6.937, *P* = 0.008; time after fight: χ^2^ = 2.263, *P* = 0.322) (Fig. 2A). Fight outcome did not influence spermatophore retention time, but the spermatophore retention time of males fought 10–12 h previously was significantly shorter than that of males fought recently (Two-way ANOVA, fight outcome: df = 1, F = 0.254, *P* = 0.615; time after fight: df = 2, F = 9.550, *P* < 0.001) (Fig. 2B).

### Effects of male dominance and body weight on the fecundity and fertilization success of females

**Figure 3:**
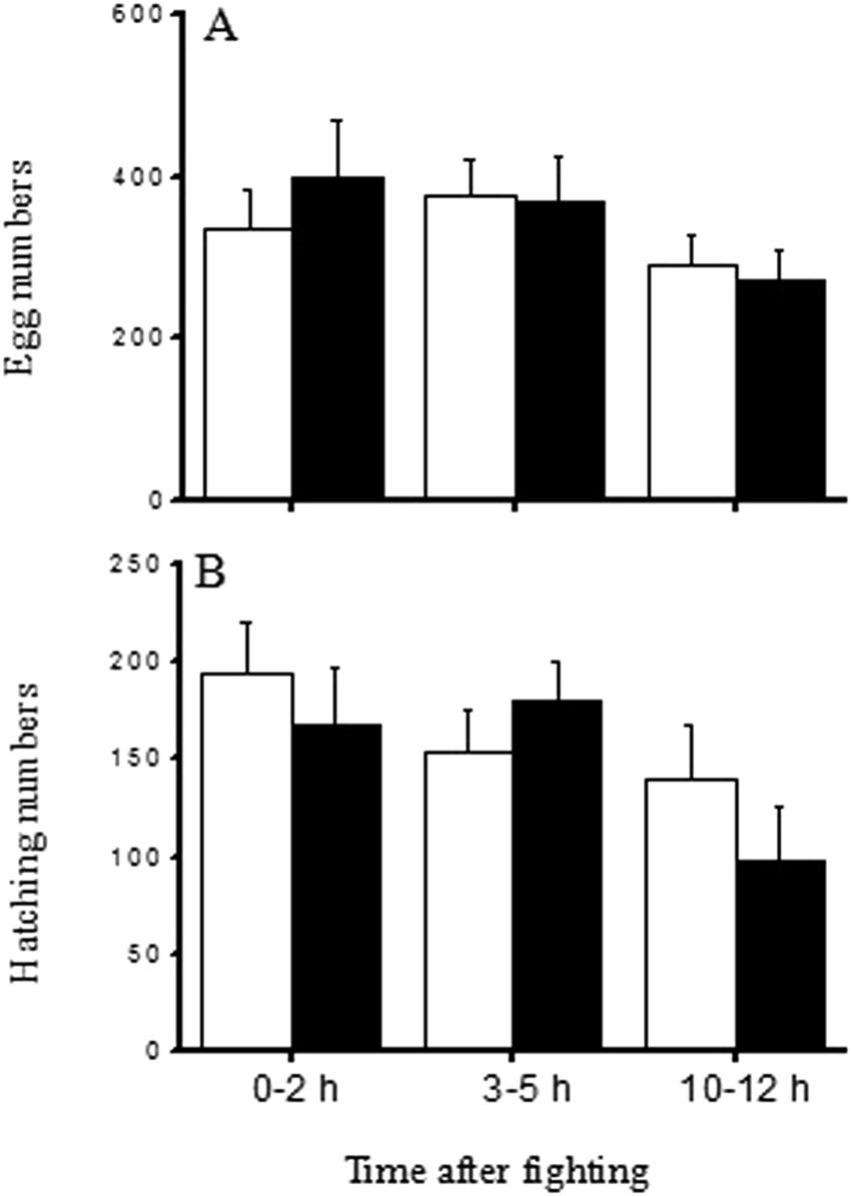
Comparison of the fecundity (**A**) and hatching number (**B**) in females that mated with winners or losers at different times after the end of fighting (empty bars: winners, solid bars: losers; n = 16–24).

**Figure 4:**
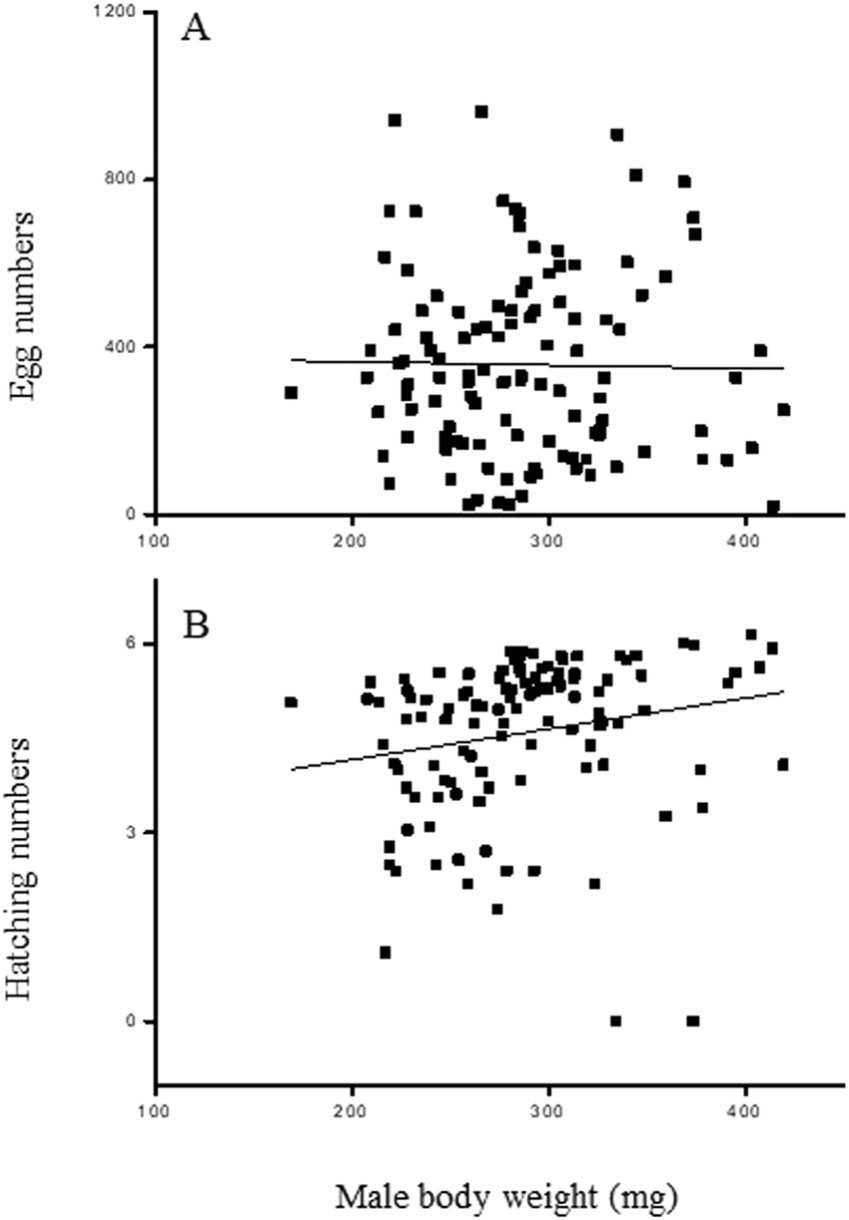
Linear regression analyses of body weight in males with fecundity (**A**) and fertilization success (**B**) in females (n = 115).

Five females died without laying any eggs, so these females were excluded from the analyses of fecundity and fertilization success. Females mated with male winners or male losers at different times after fighting laid similar amounts of eggs during the next 20 days (Two-way ANOVA, fight outcome: df = 1, F = 0.005, *P* = 0.942; time after fight: df = 2, F = 1.984, *P* = 0.055) (Fig. 3A). Less than half of the eggs hatched and the fertilization success did not differ significantly between the females mated with male winners and losers, but the fertilization success of females mated with males fought recently was higher than that of females mated with males fought 10–12 h previously (Two-way ANOVA, fight outcome: df = 1, F = 0.415, *P* = 0.521; time after fight: df = 2, F = 3.169, *P* = 0.046) (Fig. 3B). There was no significant correlation between the male body weight and the fecundity of females (linear regression analysis: F_1,113_ = 0.04, *P* = 0.85) (Fig. 4A), but the fertilization success of females was positively correlated with male body weight (linear regression analysis: F_1,113_ = 4.52, *P* = 0.04) (Fig. 4B), thereby suggesting that male body size could affect reproductive fitness during post-copulatory selection.

### Effect of dominance on the capacity for multiple mating

**Figure 5:**
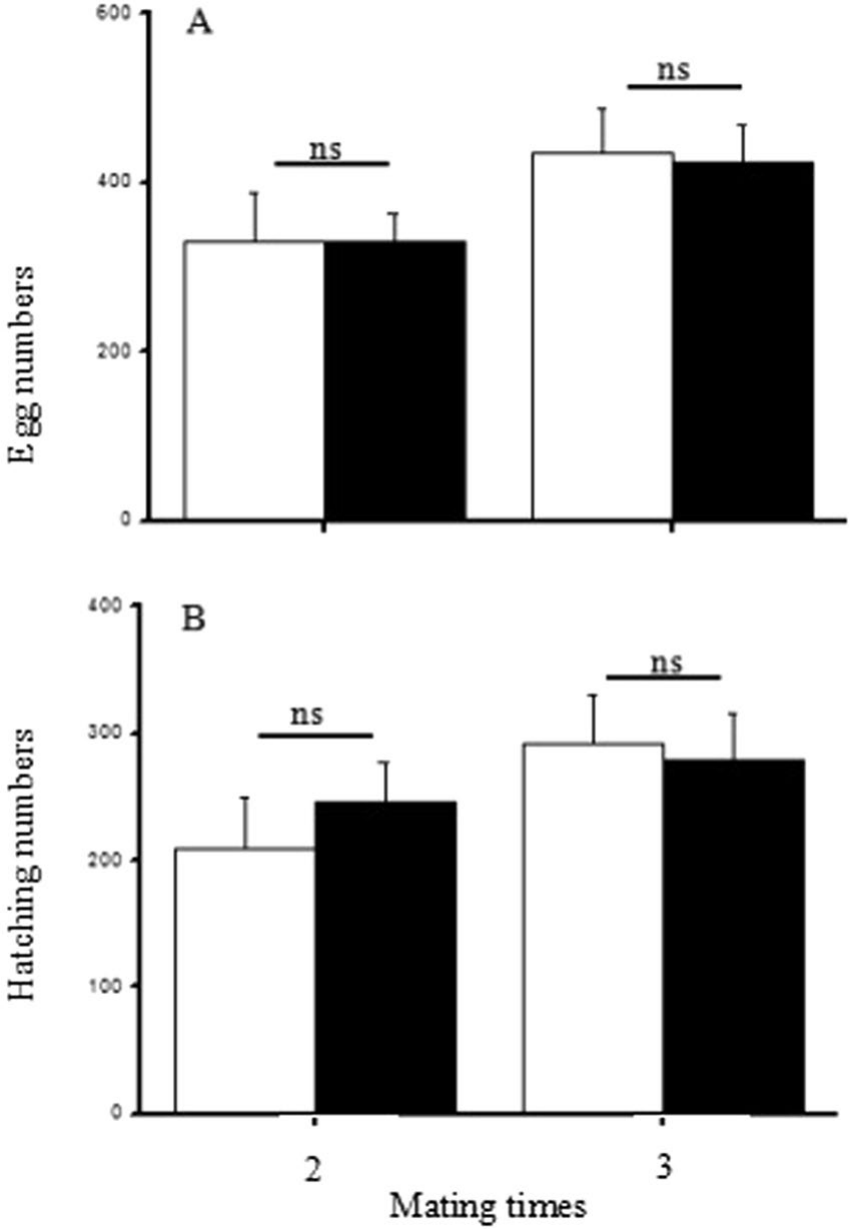
Comparison of the fecundity (**A**) and the hatching number (**B**) in females mated two and three times with winners or losers after the end of fighting (empty bars: winners, solid bars: losers; ns indicates no significant difference between winners and losers, t-test, *P* > 0.05, n = 12–26).

Fecundity did not differ significantly between females mated two or three times with male winners and male losers (*t*-test: two copulations: t_33_ = 0.16, *P* = 0.87; three copulations: t_19_ = −0.01, *P* = 0.99) (Fig. 5A). About two-thirds of the eggs hatched successfully and there were no significant differences in the fertilization success of females that mated two or three times with male winners and male losers (*t*-test: two copulations: t_33_ = 0.24, *P* = 0.81; three copulations: t_19_ = −0.71, *P* = 0.48)(Fig. 5B). These results indicate that dominance did not affect the capacity for multiple mating with males.

## Discussion

Courtship songs are a primary component of the mating behavior by male crickets and females usually do not mate with males without courtship songs. In this study, we found that losing a fight did not affect the production of courtship songs by male crickets *V*. *aspersus*, but winning a fight could enhance the courtship songs of males. Similarly, in crickets *Gryllus bimaculatus*, the courtship behavior of male losers recovered when male winners were absent and male winners were more sensitive to the initiation of courtship songs when stimulated by female wings where they required a shorter time to initiate courtship songs when stimulated by females^23^. Interestingly, this winning effect was only observed during the first 2 h after fighting, but not during the periods 3–5 h and 10–12 h after fighting, thereby suggesting that the winning experience only briefly enhanced the courtship behavior of males. Activation of a specific motor pattern briefly affects an unrelated subsequent behaviour has been reported in other species. For example, flying could briefly activated octopamine nervous system which promotes male aggression^20,24^. Behavioural modulation of motivational aspects of brain function may yield new insight as to how and why evolutionary adaptation has connected behaviours that were previously unrelated.

Pre-sexual selection is one of the primary forces that drive the evolution of extravagant phenotypic traits in males. Some studies have shown that females prefer to mate with male winners than losers. For example, male winners of the house cricket *Acheta domesticus* are significantly more likely to court females than male losers^25^. There is great diversity in the signals employed by females to discriminate male winners from losers. In some insects, females discriminate the winners from losers based on pheromones^26^. Other studies have shown that females incite male competition to facilitate mate choice^27,28^, thereby suggesting that females may discriminate winners from losers by watching the males fighting. By contrast, female *Teleogryllus commodus* do not prefer to mate with males that win fights^11^. Our results showed that female crickets *V*. *aspersus* mated more often with male winners during the first 2 h after fighting when the male winners were more likely to produce courtship songs than losers, suggesting that fight outcome affects reproductive fitness of males during pre-copulatory selection. However, females mated with equal number of male winners and losers during 3–5 and 10–12 hours after fighting. It appears that this difference in the courtship singing behavior may cause females *V*. *aspersus* to mate with more male winners than losers.

Our results also showed that reproductive fitness in post-copulatory selection was affected by male body weight but not by male dominance. It seems that females may prefer to store sperms of larger males, or larger males transfer more sperm or seminal fluid products that increase fertilization success. In the cricket *G*. *bimaculatus*, females that mated with potentially more dominant males laid more eggs, which suggests that the fighting ability of males may have a positive effect on post-copulatory female selection^29^. Thomas and Simmons^26^ also found that subordinate male crickets *Teleogryllus oceanicus* produced lower quality ejaculates than dominant males and they sired less offspring when competing for fertilization. In these previous studies, males were forced to fight several rounds, so the body quality of the males that won all of their fights may have been much higher than that of the males that lost all of their fights. In the current study, the males only fought once, so the difference in quality between male winners and losers might not have been sufficiently large. Alternatively, the fighting ability of males can be affected by many factors, including their body size, health status, reproductive development, weapon size, and personality^30–33^. Some traits may also be positively correlated with the sperm number or quality, thereby directly or indirectly increasing the reproductive fitness of females. For example, sperm number was significantly higher in larger males in mosquitofish^34^ and crickets^35^. However, some other traits may be negatively correlated with investment in sperm number or quality. For example, in two species of hissing cockroaches, *Gromphadorhina oblongonota* and *Aeluropoda insignis*, individuals invest more heavily in weapon length at the expense of the testes mass^3^. Therefore, mating with a dominant male might not increase the reproductive fitness in some circumstances and females should prefer males that develop better traits correlated with sperm number or quality after copulation.

Interestingly, the time after a fight also influenced mating behavior of both males and females. Males after a recent fight required more time to initiate courtship songs, but females retained the attached spermatophores of males fought recently longer than males fought 10–12 h previously. It seems that males may need more time to produce larger spermatophores when they recently experienced a fight. Similarly, the presence of potential competitors causes males to increase sperm number in other cricket species, *G*. *bimaculatus* and *Gryllodes sigillatus*, and cockroach *Nauphoeta cinerea*^36,37^. However, we did not compare the spermatophore size between males fought recently and males fought 10–12 h previously, so this inference needs to be further tested.

## Methods

### Experimental individuals

Crickets *V*. *aspersus* were collected from Hainan Province in China, and reared in our laboratory under the condition of LD 16:8 h, 30 °C. Nymphs were kept in groups (50 nymphs/container), and provided with artificial insect feed (DaRui Co., Changsha, China) and water. Male adults were kept separately until they were used in the following experiments. The fighting trials were conducted on day 8–9 of adulthood, because males were sexually matured at 7 days after molting to adulthood^38^.

### Effects of dominance on the courtship behavior of males and female mating choice

Body weight of males were measured with a digital scale (0.0001 g) and about 200 pairs of males matched by body weight (difference of less than 2%) were used in fighting experiment. To discriminate each male within the pair, the males were marked with red or black color on their pronotum using a marker pen. Each pair of males were allowed to fight in a transparent round plastic container following the method described in Zeng *et al*.^22^. Dominance was established when one male sung and chased around the other. Fighting trials were observed within a period of 30 min and those where the clear establishment of dominance did not occur were excluded. After fighting, the winners and losers were returned to their own containers and 120 pairs of winners and losers were used to conduct mating trials. Mating trials were performed over three periods comprising 0–2 h, 3–5 h, and 10–12 h after fighting, and 40 pairs of winners and losers were used for each time period. In each mating trial, a sexually mature virgin female (15 days after adulthood) was introduced into the container with the male and the mating behavior was observed for 2 h. We recorded the courtship singing rate and time required to initiate singing by males, as well as the mating rates of females and the spermatophore retention time.

### Effects of male dominance and body weight on fecundity and fertilization success of females

To examine whether the fecundity and fertilization success of females differed when mated with male winners or losers, the females that mated successfully in mating trials were housed separately and provided with ovipositional substrates. Eggs laid over the following 20 days were collected and hatched under 25 °C. After no further eggs hatched over 10 successive days, the remaining eggs were observed and counted under a microscope (Leica, Germany). Male body weight is positively correlated with spermatophore size, so we also analyzed the correlations between male body weight and female fecundity and fertilization success.

### Effect of dominance on the capacity for multiple mating

Previous study has shown that this species is polygamous and that multiple copulation increases the fertilization success of females^39^. Therefore, we examined the effects of dominance on the fecundity and fertilization success of females when mated two or three times. We introduced 120 pairs of males matched by weight into a transparent round plastic arena to fight and establish clear dominance. Next, 60 pairs of winners and losers were kept separately with a female for 4 h to copulate twice, and 60 pairs of winners and losers were kept separately with a female for 6 h to copulate three times. After the mating trial, the females that mated two or three times were housed separately and provided with ovipositional substrates. Eggs laid over the following 20 days were collected and hatched at 25 °C. After no further eggs hatched over 10 successive days, the remaining eggs were observed and counted under a microscope.

### Data analysis

Effects of fight outcome and time after a fight on courtship singing rate and mating rate were analyzed by Generalized Linear Models (GLM) with binomial errors. Effects of fight outcome and time after a fight on the time required to initiate courtship songs, spermatophore retention time, fecundity, and fertilization success results were analyzed by Two-way ANOVA. Comparisons of fecundity and fertilization between females mated multiple times with male winners and male losers were analyzed by Student’s *t*-test. Correlations between male body weight and the fecundity or fertilization success (data were log transformed) of females were analyzed by linear regression.
